# Supplementary material for: Are carbon emissions trading and green financial instruments synergistic? -Comprehensive quantitative research based on content analysis
Source: PLoS One. 2024 Mar 7;19(3):e0298601. doi: 10.1371/journal.pone.0298601 (PMC10919601; doi:10.1371/journal.pone.0298601)
Supplement: S1 Data — (ZIP) [file pone.0298601.s001.zip › Data/地级市Z_F工作报告.zip!/─┌├╔╣┼/║⌠║═║╞╠╪╩╨/└┤╫╘╣1⁄2╓┌║┼í╛┬φ┐╦╩2╛▌═°í┐_2019.docx]

各位代表:

现在，我代表国务院，向大会报告政府工作，请予审议，并请全国政协委员提出意见。

一、2018年工作回顾

过去一年是全面贯彻党的十九大精神开局之年，是本届政府依法履职第一年。我国发展面临多年少有的国内外复杂严峻形势，经济出现新的下行压力。在以习近平同志为核心的党中央坚强领导下，全国各族人民以习近平新时代中国特色社会主义思想为指导，砥砺奋进，攻坚克难，完成全年经济社会发展主要目标任务，决胜全面建成小康社会又取得新的重大进展。

——经济运行保持在合理区间。国内生产总值增长6.6%，总量突破90万亿元。经济增速与用电、货运等实物量指标相匹配。居民消费价格上涨2.1%。国际收支基本平衡。城镇新增就业1361万人、调查失业率稳定在5%左右的较低水平。近14亿人口的发展中大国，实现了比较充分就业。

——经济结构不断优化。消费拉动经济增长作用进一步增强。服务业对经济增长贡献率接近60%，高技术产业、装备制造业增速明显快于一般工业，农业再获丰收。单位国内生产总值能耗下降3.1%。质量和效益继续提升。

——发展新动能快速成长。嫦娥四号等一批重大科技创新成果相继问世。新兴产业蓬勃发展，传统产业加快转型升级。大众创业万众创新深入推进，日均新设企业超过1.8万户，市场主体总量超过1亿户。新动能正在深刻改变生产生活方式、塑造中国发展新优势。

——改革开放取得新突破。国务院及地方政府机构改革顺利实施。重点领域改革迈出新的步伐，市场准入负面清单制度全面实行，简政放权、放管结合、优化服务改革力度加大，营商环境国际排名大幅上升。对外开放全方位扩大，共建“一带一路”取得重要进展。首届中国国际进口博览会成功举办，海南自贸试验区启动建设。货物进出口总额超过30万亿元，实际使用外资1383亿美元、稳居发展中国家首位。

——三大攻坚战开局良好。防范化解重大风险，宏观杠杆率趋于稳定，金融运行总体平稳。精准脱贫有力推进，农村贫困人口减少1386万，易地扶贫搬迁280万人。污染防治得到加强，细颗粒物(PM2.5)浓度继续下降，生态文明建设成效显著。

——人民生活持续改善。居民人均可支配收入实际增长6.5%。提高个人所得税起征点，设立6项专项附加扣除。加大基本养老、基本医疗等保障力度，资助各类学校家庭困难学生近1亿人次。棚户区住房改造620多万套，农村危房改造190万户。城乡居民生活水平又有新提高。

我们隆重庆祝改革开放40周年，深刻总结改革开放的伟大成就和宝贵经验，郑重宣示在新时代将改革开放进行到底的坚定决心，激励全国各族人民接续奋斗，再创新的历史伟业。

回顾过去一年，成绩来之不易。我们面对的是深刻变化的外部环境。经济全球化遭遇波折，多边主义受到冲击，国际金融市场震荡，特别是中美经贸摩擦给一些企业生产经营、市场预期带来不利影响。我们面对的是经济转型阵痛凸显的严峻挑战。新老矛盾交织，周期性、结构性问题叠加，经济运行稳中有变、变中有忧。我们面对的是两难多难问题增多的复杂局面。实现稳增长、防风险等多重目标，完成经济社会发展等多项任务，处理好当前与长远等多种关系，政策抉择和工作推进的难度明显加大。经过全国上下共同努力，我国经济发展在高基数上总体平稳、稳中有进，社会大局保持稳定。这再次表明，在中国共产党领导下，中国人民有战胜任何艰难险阻的勇气、智慧和力量，中国的发展没有过不去的坎。

一年来，我们深入贯彻以习近平同志为核心的党中央决策部署，坚持稳中求进工作总基调，统筹稳增长、促改革、调结构、惠民生、防风险，稳妥应对中美经贸摩擦，着力稳就业、稳金融、稳外贸、稳外资、稳投资、稳预期，主要做了以下工作。

一是创新和完善宏观调控，经济保持平稳运行。面对新情况新变化，我们坚持不搞“大水漫灌”式强刺激，保持宏观政策连续性稳定性，在区间调控基础上加强定向、相机调控，主动预调、微调。坚持实施积极的财政政策，着力减税降费、补短板调结构。下调增值税税率，扩大享受税收优惠小微企业范围，出台鼓励研发创新等税收政策。全年为企业和个人减税降费约1.3万亿元。优化财政支出结构，盘活财政存量资金，重点领域支出得到保障。坚持实施稳健的货币政策，引导金融支持实体经济。针对融资难融资贵问题，先后4次降低存款准备金率，多措并举缓解民营和小微企业资金紧张状况，融资成本上升势头得到初步遏制。及时应对股市、债市异常波动，人民币汇率基本稳定，外汇储备保持在3万亿美元以上。

二是扎实打好三大攻坚战，重点任务取得积极进展。制定并有序实施三大攻坚战三年行动方案。稳步推进结构性去杠杆，稳妥处置金融领域风险，防控地方政府债务风险，改革完善房地产市场调控机制。深入推进精准脱贫，加强扶贫力量，加大资金投入，强化社会帮扶，贫困地区自我发展能力稳步提高。全面开展蓝天、碧水、净土保卫战。优化能源和运输结构。稳妥推进北方地区“煤改气”“煤改电”。全面建立河长制、湖长制。化肥农药使用量实现双下降。加强生态环保督察执法。积极应对气候变化。

三是深化供给侧结构性改革，实体经济活力不断释放。加大“破、立、降”力度。推进钢铁、煤炭行业市场化去产能。实施稳投资举措，制造业投资、民间投资增速明显回升。出台促进居民消费政策。全面推进“互联网+”，运用新技术新模式改造传统产业。深入推进简政减税减费。取消一批行政许可事项，“证照分离”改革在全国推开，企业开办时间大幅压缩，工业生产许可证种类压减三分之一以上。“双随机、一公开”监管全面实施。清理规范各类涉企收费，推动降低用能、用网和物流等成本。深化“互联网+政务服务”，各地探索推广一批有特色的改革举措，企业和群众办事便利度不断提高。

四是深入实施创新驱动发展战略，创新能力和效率进一步提升。大力优化创新生态，调动各类创新主体积极性。深化科技管理体制改革，推进关键核心技术攻关，加强重大科技基础设施、科技创新中心等建设。强化企业技术创新主体地位，将提高研发费用加计扣除比例政策扩大至所有企业。制定支持双创深入发展的政策措施。技术合同成交额增长30%以上。科技进步贡献率提高到58.5%。

五是加大改革开放力度，发展动力继续增强。深化国资国企改革，国有企业优化重组、提质增效取得新进展。针对民营企业发展遇到的困难和问题，千方百计帮助解忧纾困。推进财税体制改革，预算绩效管理改革全面启动。改革金融监管体制，完善利率、汇率市场化形成机制。农业农村、社会事业、生态环保等领域改革不断深化。推出对外开放一系列重大举措。共建“一带一路”引领效应持续释放，同沿线国家的合作机制不断健全，经贸合作和人文交流加快推进。出台稳外贸政策，货物通关时间压缩一半以上。下调部分商品进口关税，关税总水平由9.8%降至7.5%。新设一批跨境电商综合试验区。复制推广自贸试验区改革经验。大幅压缩外资准入负面清单，扩大金融、汽车等行业开放，一批重大外资项目落地，新设外资企业增长近70%。

六是统筹城乡区域发展，良性互动格局加快形成。乡村振兴战略有力实施，粮食总产量保持在1.3万亿斤以上。新型城镇化扎实推进，近1400万农业转移人口在城镇落户。推进西部开发、东北振兴、中部崛起、东部率先发展，出台一批改革创新举措。京津冀协同发展取得明显进展，长江经济带生态优先、绿色发展格局不断巩固。粤港澳大湾区规划建设迈出实质性步伐，港珠澳大桥建成通车。加大对革命老区、民族地区、边疆地区、贫困地区改革发展支持力度。新增高速铁路运营里程4100公里，新建改建高速公路6000多公里、农村公路30多万公里。城乡区域发展协调性持续增强。

七是坚持在发展中保障和改善民生，改革发展成果更多更公平惠及人民群众。针对外部环境变化给就业带来的影响，及时出台稳就业举措。大力推动义务教育教师工资待遇政策落实，加强乡村小规模学校和乡镇寄宿制学校建设，促进高等教育内涵式发展。建立企业职工基本养老保险基金中央调剂制度，提高退休人员基本养老金，城乡居民基础养老金最低标准从每月70元提高到88元。继续提高优抚、低保等标准，残疾人“两项补贴”惠及所有符合条件人员。加强退役军人服务管理工作，维护退役军人合法权益。深化医疗、医保、医药联动改革。稳步推进分级诊疗。提高居民基本医保补助标准和大病保险报销比例。加快新药审评审批改革，17种抗癌药大幅降价并纳入国家医保目录。加快推进文化惠民工程，持续加强基层公共文化服务。全民健身蓬勃开展，体育健儿在国际大赛上再创佳绩。

八是推进法治政府建设和治理创新，保持社会和谐稳定。提请全国人大常委会审议法律议案18件，制定修订行政法规37部。改革调整政府机构设置和职能配置。深入开展国务院大督查，推动改革发展政策和部署落实。发挥审计监督作用。改革完善城乡基层治理。创新信访工作方式。改革和加强应急管理，及时有效应对重大自然灾害，生产安全事故总量和重特大事故数量继续下降。加强食品药品安全监管，严厉查处长春长生公司等问题疫苗案件。健全国家安全体系。强化社会治安综合治理，开展扫黑除恶专项斗争，依法打击各类违法犯罪，平安中国建设取得新进展。

认真贯彻党中央全面从严治党战略部署，加强党风廉政建设。推进“两学一做”学习教育常态化制度化。严格落实中央八项规定及其实施细则精神，坚定不移纠正“四风”。严肃查处各类违法违规行为，惩处腐败分子，反腐败斗争取得压倒性胜利。

过去一年，中国特色大国外交取得新成就。成功举办博鳌亚洲论坛年会、上合组织青岛峰会、中非合作论坛北京峰会等重大主场外交活动。习近平主席等国家领导人出访多国，出席亚太经合组织领导人非正式会议、二十国集团领导人峰会、金砖国家领导人会晤、亚欧首脑会议、东亚合作领导人系列会议等重大活动。同主要大国关系总体稳定，同周边国家关系全面发展，同发展中国家团结合作纽带更加牢固。推动构建新型国际关系，推动构建人类命运共同体。坚定维护国家主权、安全、发展利益。经济外交、人文交流成果丰硕。中国致力于促进世界和平与发展，作出了世人共睹的重要贡献。

各位代表!

过去一年取得的成绩，是以习近平同志为核心的党中央坚强领导的结果，是习近平新时代中国特色社会主义思想科学指引的结果，是全党全军全国各族人民团结奋斗的结果。我代表国务院，向全国各族人民，向各民主党派、各人民团体和各界人士，表示诚挚感谢!向香港特别行政区同胞、澳门特别行政区同胞、台湾同胞和海外侨胞，表示诚挚感谢!向关心和支持中国现代化建设的各国政府、国际组织和各国朋友，表示诚挚感谢!

思危方能居安。在充分肯定成绩的同时，要清醒看到我国发展面临的问题和挑战。世界经济增速放缓，保护主义、单边主义加剧，国际大宗商品价格大幅波动，不稳定不确定因素明显增加，外部输入性风险上升。国内经济下行压力加大，消费增速减慢，有效投资增长乏力。实体经济困难较多，民营和小微企业融资难融资贵问题尚未有效缓解，营商环境与市场主体期待还有较大差距。自主创新能力不强，关键核心技术短板问题凸显。一些地方财政收支矛盾较大。金融等领域风险隐患依然不少。深度贫困地区脱贫攻坚困难较多。生态保护和污染防治任务仍然繁重。在教育、医疗、养老、住房、食品药品安全、收入分配等方面，群众还有不少不满意的地方。去年还发生了多起公共安全事件和重大生产安全事故，教训极其深刻。政府工作存在不足，一些改革发展举措落实不到位，形式主义、官僚主义仍然突出，督查检查考核过多过频、重留痕轻实绩，加重基层负担。少数干部懒政怠政。一些领域腐败问题仍然多发。我们一定要直面问题和挑战，勇于担当，恪尽职守，竭尽全力做好工作，决不辜负人民期待!

二、2019年经济社会发展总体要求和政策取向

今年是新中国成立70周年，是全面建成小康社会、实现第一个百年奋斗目标的关键之年。做好政府工作，要在以习近平同志为核心的党中央坚强领导下，以习近平新时代中国特色社会主义思想为指导，全面贯彻党的十九大和十九届二中、三中全会精神，统筹推进“五位一体”总体布局，协调推进“四个全面”战略布局，坚持稳中求进工作总基调，坚持新发展理念，坚持推动高质量发展，坚持以供给侧结构性改革为主线，坚持深化市场化改革、扩大高水平开放，加快建设现代化经济体系，继续打好三大攻坚战，着力激发微观主体活力，创新和完善宏观调控，统筹推进稳增长、促改革、调结构、惠民生、防风险、保稳定工作，保持经济运行在合理区间，进一步稳就业、稳金融、稳外贸、稳外资、稳投资、稳预期，提振市场信心，增强人民群众获得感、幸福感、安全感，保持经济持续健康发展和社会大局稳定，为全面建成小康社会收官打下决定性基础，以优异成绩庆祝中华人民共和国成立70周年。

综合分析国内外形势，今年我国发展面临的环境更复杂更严峻，可以预料和难以预料的风险挑战更多更大，要做好打硬仗的充分准备。困难不容低估，信心不可动摇，干劲不能松懈。我国发展仍处于重要战略机遇期，拥有足够的韧性、巨大的潜力和不断迸发的创新活力，人民群众追求美好生活的愿望十分强烈。我们有战胜各种困难挑战的坚定意志和能力，经济长期向好趋势没有也不会改变。

今年经济社会发展的主要预期目标是:国内生产总值增长6%-6.5%;城镇新增就业1100万人以上，城镇调查失业率5.5%左右，城镇登记失业率4.5%以内;居民消费价格涨幅3%左右;国际收支基本平衡，进出口稳中提质;宏观杠杆率基本稳定，金融财政风险有效防控;农村贫困人口减少1000万以上，居民收入增长与经济增长基本同步;生态环境进一步改善，单位国内生产总值能耗下降3%左右，主要污染物排放量继续下降。上述主要预期目标，体现了推动高质量发展要求，符合我国发展实际，与全面建成小康社会目标相衔接，是积极稳妥的。实现这些目标，需要付出艰苦努力。

要正确把握宏观政策取向，继续实施积极的财政政策和稳健的货币政策，实施就业优先政策，加强政策协调配合，确保经济运行在合理区间，促进经济社会持续健康发展。

积极的财政政策要加力提效。今年赤字率拟按2.8%安排，比去年预算高0.2个百分点;财政赤字2.76万亿元，其中中央财政赤字1.83万亿元，地方财政赤字9300亿元。适度提高赤字率，综合考虑了财政收支、专项债券发行等因素，也考虑为应对今后可能出现的风险留出政策空间。今年财政支出超过23万亿元，增长6.5%。中央对地方均衡性转移支付增长10.9%。改革完善县级基本财力保障机制，缓解困难地区财政运转压力，决不让基本民生保障出问题。

稳健的货币政策要松紧适度。广义货币M2和社会融资规模增速要与国内生产总值名义增速相匹配，以更好满足经济运行保持在合理区间的需要。在实际执行中，既要把好货币供给总闸门，不搞“大水漫灌”，又要灵活运用多种货币政策工具，疏通货币政策传导渠道，保持流动性合理充裕，有效缓解实体经济特别是民营和小微企业融资难融资贵问题，防范化解金融风险。深化利率市场化改革，降低实际利率水平。完善汇率形成机制，保持人民币汇率在合理均衡水平上的基本稳定。

就业优先政策要全面发力。就业是民生之本、财富之源。今年首次将就业优先政策置于宏观政策层面，旨在强化各方面重视就业、支持就业的导向。当前和今后一个时期，我国就业总量压力不减、结构性矛盾凸显，新的影响因素还在增加，必须把就业摆在更加突出位置。稳增长首要是为保就业。今年城镇新增就业要在实现预期目标的基础上，力争达到近几年的实际规模，既保障城镇劳动力就业，也为农业富余劳动力转移就业留出空间。只要就业稳、收入增，我们就更有底气。

要继续坚持以供给侧结构性改革为主线，在“巩固、增强、提升、畅通”八个字上下功夫。更多采取改革的办法，更多运用市场化、法治化手段，巩固“三去一降一补”成果，增强微观主体活力，提升产业链水平，畅通国民经济循环，推动经济高质量发展。

要继续打好三大攻坚战，精准发力、务求实效。防范化解重大风险要强化底线思维，坚持结构性去杠杆，防范金融市场异常波动，稳妥处理地方政府债务风险，防控输入性风险。精准脱贫要坚持现行标准，聚焦深度贫困地区和特殊贫困群体，加大攻坚力度，提高脱贫质量。污染防治要聚焦打赢蓝天保卫战等重点任务，统筹兼顾、标本兼治，使生态环境质量持续改善。

做好今年政府工作，要注重把握好以下关系。一要统筹好国内与国际的关系，凝心聚力办好自己的事。我国仍处于并将长期处于社会主义初级阶段，仍是世界最大发展中国家。发展是解决我国一切问题的基础和关键，必须牢牢扭住经济建设这个中心，毫不动摇坚持发展是硬道理、发展应该是科学发展和高质量发展的战略思想，不断解放和发展社会生产力。在国际形势复杂多变的背景下，我们要保持战略定力，按确定的目标和部署推进工作，更好利用国际国内两个市场两种资源，敢于应对挑战，善于化危为机，牢牢把握发展主动权。二要平衡好稳增长与防风险的关系，确保经济持续健康发展。长期积累的诸多风险隐患必须加以化解，但要遵循规律，讲究方式方法，按照坚定、可控、有序、适度要求，在发展中逐步化解，坚决避免发生系统性、区域性风险。在当前经济下行压力加大情况下，出台政策和工作举措要有利于稳预期、稳增长、调结构，防控风险要把握好节奏和力度，防止紧缩效应叠加放大，决不能让经济运行滑出合理区间。同时，也不能只顾眼前，采取损害长期发展的短期强刺激政策，产生新的风险隐患。三要处理好政府与市场的关系，依靠改革开放激发市场主体活力。只要市场主体有活力，就能增强内生发展动力、顶住经济下行压力。要大力推进改革开放，加快建立统一开放、竞争有序的现代市场体系，放宽市场准入，加强公正监管，打造法治化、国际化、便利化的营商环境，让各类市场主体更加活跃。从根本上说，市场活力和社会创造力源于亿万人民积极性的发挥。要坚持以人民为中心的发展思想，尽力而为、量力而行，切实保障基本民生，推动解决重点民生问题，促进社会公平正义，让人民过上好日子。中国人民勤劳智慧，具有无限的创新创造潜能，只要充分释放出来，中国的发展就一定会有更为广阔空间。

三、2019年政府工作任务

今年经济社会发展任务重、挑战多、要求高。我们要突出重点、把握关键，扎实做好各项工作。

(一)继续创新和完善宏观调控，确保经济运行在合理区间。坚持以市场化改革的思路和办法破解发展难题，发挥好宏观政策逆周期调节作用，丰富和灵活运用财政、货币、就业政策工具，增强调控前瞻性、针对性和有效性，为经济平稳运行创造条件。

实施更大规模的减税。普惠性减税与结构性减税并举，重点降低制造业和小微企业税收负担。深化增值税改革，将制造业等行业现行16%的税率降至13%，将交通运输业、建筑业等行业现行10%的税率降至9%，确保主要行业税负明显降低;保持6%一档的税率不变，但通过采取对生产、生活性服务业增加税收抵扣等配套措施，确保所有行业税负只减不增，继续向推进税率三档并两档、税制简化方向迈进。抓好年初出台的小微企业普惠性减税政策落实。这次减税，着眼“放水养鱼”、增强发展后劲并考虑财政可持续，是减轻企业负担、激发市场活力的重大举措，是完善税制、优化收入分配格局的重要改革，是宏观政策支持稳增长、保就业、调结构的重大抉择。

明显降低企业社保缴费负担。下调城镇职工基本养老保险单位缴费比例，各地可降至16%。稳定现行征缴方式，各地在征收体制改革过程中不得采取增加小微企业实际缴费负担的做法，不得自行对历史欠费进行集中清缴。继续执行阶段性降低失业和工伤保险费率政策。今年务必使企业特别是小微企业社保缴费负担有实质性下降。加快推进养老保险省级统筹改革，继续提高企业职工基本养老保险基金中央调剂比例、划转部分国有资本充实社保基金。我们既要减轻企业缴费负担，又要保障职工社保待遇不受影响、养老金合理增长并按时足额发放，使社保基金可持续、企业与职工同受益。

确保减税降费落实到位。减税降费直击当前市场主体的痛点和难点，是既公平又有效率的政策。全年减轻企业税收和社保缴费负担近2万亿元。这会给各级财政带来很大压力。为支持企业减负，各级政府要过紧日子，想方设法筹集资金。中央财政要开源节流，增加特定国有金融机构和央企上缴利润，一般性支出压减5%以上、“三公”经费再压减3%左右，长期沉淀资金一律收回。地方政府也要主动挖潜，大力优化支出结构，多渠道盘活各类资金和资产。我们要切实让市场主体特别是小微企业有明显减税降费感受，坚决兑现对企业和社会的承诺，困难再多也一定要把这件大事办成办好。

着力缓解企业融资难融资贵问题。改革完善货币信贷投放机制，适时运用存款准备金率、利率等数量和价格手段，引导金融机构扩大信贷投放、降低贷款成本，精准有效支持实体经济，不能让资金空转或脱实向虚。加大对中小银行定向降准力度，释放的资金全部用于民营和小微企业贷款。支持大型商业银行多渠道补充资本，增强信贷投放能力，鼓励增加制造业中长期贷款和信用贷款。今年国有大型商业银行小微企业贷款要增长30%以上。清理规范银行及中介服务收费。完善金融机构内部考核机制，激励加强普惠金融服务，切实使中小微企业融资紧张状况有明显改善，综合融资成本必须有明显降低。

有效发挥地方政府债券作用。今年拟安排地方政府专项债券2.15万亿元，比去年增加8000亿元，为重点项目建设提供资金支持，也为更好防范化解地方政府债务风险创造条件。合理扩大专项债券使用范围。继续发行一定数量的地方政府置换债券，减轻地方利息负担。鼓励采取市场化方式，妥善解决融资平台到期债务问题，不能搞“半拉子”工程。

多管齐下稳定和扩大就业。扎实做好高校毕业生、退役军人、农民工等重点群体就业工作，加强对城镇各类就业困难人员的就业帮扶。对招用农村贫困人口、城镇登记失业半年以上人员的各类企业，三年内给予定额税费减免。加强对灵活就业、新就业形态的支持。坚决防止和纠正就业中的性别和身份歧视。实施职业技能提升行动，从失业保险基金结余中拿出1000亿元，用于1500万人次以上的职工技能提升和转岗转业培训。健全技术工人职业发展机制和政策。加快发展现代职业教育，既有利于缓解当前就业压力，也是解决高技能人才短缺的战略之举。改革完善高职院校考试招生办法，鼓励更多应届高中毕业生和退役军人、下岗职工、农民工等报考，今年大规模扩招100万人。扩大高职院校奖助学金覆盖面、提高补助标准，加快学历证书和职业技能等级证书互通衔接。改革高职院校办学体制，加强师资队伍建设，提高办学质量。引导一批普通本科高校转为应用型大学。中央财政大幅增加对高职院校的投入，地方财政也要加强支持。设立中等职业教育国家奖学金。支持企业和社会力量兴办职业教育，加快产教融合实训基地建设。我们要以现代职业教育的大改革大发展，加快培养国家发展急需的各类技术技能人才，让更多青年凭借一技之长实现人生价值，让三百六十行人才荟萃、繁星璀璨。

(二)激发市场主体活力，着力优化营商环境。我国有上亿市场主体，而且还在不断增加。把市场主体的活跃度保持住、提上去，是促进经济平稳增长的关键所在。要深化“放管服”改革，降低制度性交易成本，下硬功夫打造好发展软环境。

以简审批优服务便利投资兴业。市场配置资源是最有效率的形式。要进一步缩减市场准入负面清单，推动“非禁即入”普遍落实。政府要坚决把不该管的事项交给市场，最大限度减少对资源的直接配置，审批事项应减尽减，确需审批的要简化流程和环节，让企业多用时间跑市场、少费功夫跑审批。今年，要对所有涉企经营许可事项实行“证照分离”改革，使企业更便捷拿到营业执照并尽快正常运营，坚决克服“准入不准营”的现象;在全国推开工程建设项目审批制度改革，使全流程审批时间大幅缩短。继续压缩专利审查和商标注册时间。推行网上审批和服务，抓紧建成全国一体化在线政务服务平台，加快实现一网通办、异地可办，使更多事项不见面办理，确需到现场办的要“一窗受理、限时办结”“最多跑一次”。持续开展“减证便民”改革行动，不能让繁琐证明来回折腾企业和群众。建立政务服务“好差评”制度，服务绩效由企业和群众来评判。政府部门做好服务是本分，服务不好是失职。

以公正监管促进公平竞争。公平竞争是市场经济的核心，公正监管是公平竞争的保障。改革完善公平竞争审查和公正监管制度，加快清理妨碍统一市场和公平竞争的各种规定和做法。政简易从。规则越简约透明，监管越有力有效。国家层面重在制定统一的监管规则和标准，地方政府要把主要力量放在公正监管上。推进“双随机、一公开”跨部门联合监管，推行信用监管和“互联网+监管”改革，优化环保、消防、税务、市场监管等执法方式，对违法者依法严惩、对守法者无事不扰。深化综合行政执法改革，清理规范行政处罚事项，坚决治理多头检查、重复检查。对监管者也要强监管、立规矩，决不允许搞选择性执法、任性执法，决不允许刁难企业和群众。依法打击制售假冒伪劣商品等违法行为，让严重违法者付出付不起的代价。完善失信联合惩戒机制，促进各类市场主体守法诚信经营。用公正监管管出公平、管出效率、管出活力。

以改革推动降低涉企收费。深化电力市场化改革，清理电价附加收费，降低制造业用电成本，一般工商业平均电价再降低10%。深化收费公路制度改革，推动降低过路过桥费用，治理对客货运车辆不合理审批和乱收费、乱罚款。两年内基本取消全国高速公路省界收费站，实现不停车快捷收费，减少拥堵、便利群众。取消或降低一批铁路、港口收费。专项治理中介服务收费。继续清理规范行政事业性收费。加快收费清单“一张网”建设，让收费公开透明，让乱收费无处藏身。

(三)坚持创新引领发展，培育壮大新动能。发挥我国人力人才资源丰富、国内市场巨大等综合优势，改革创新科技研发和产业化应用机制，大力培育专业精神，促进新旧动能接续转换。

推动传统产业改造提升。围绕推动制造业高质量发展，强化工业基础和技术创新能力，促进先进制造业和现代服务业融合发展，加快建设制造强国。打造工业互联网平台，拓展“智能+”，为制造业转型升级赋能。支持企业加快技术改造和设备更新，将固定资产加速折旧优惠政策扩大至全部制造业领域。强化质量基础支撑，推动标准与国际先进水平对接，提升产品和服务品质，让更多国内外用户选择中国制造、中国服务。

促进新兴产业加快发展。深化大数据、人工智能等研发应用，培育新一代信息技术、高端装备、生物医药、新能源汽车、新材料等新兴产业集群，壮大数字经济。坚持包容审慎监管，支持新业态新模式发展，促进平台经济、共享经济健康成长。加快在各行业各领域推进“互联网+”。持续推动网络提速降费。开展城市千兆宽带入户示范，改造提升远程教育、远程医疗网络，推动移动网络扩容升级，让用户切实感受到网速更快更稳定。今年中小企业宽带平均资费再降低15%，移动网络流量平均资费再降低20%以上，在全国实行“携号转网”，规范套餐设置，使降费实实在在、消费者明明白白。

提升科技支撑能力。加大基础研究和应用基础研究支持力度，强化原始创新，加强关键核心技术攻关。抓紧布局国家实验室，重组国家重点实验室体系。完善重大科技项目组织管理。健全以企业为主体的产学研一体化创新机制，支持企业牵头实施重大科技项目。加快建设科技创新资源开放共享平台，强化对中小企业的技术创新服务。扩大国际创新合作。全面加强知识产权保护，健全知识产权侵权惩罚性赔偿制度，促进发明创造和转化运用。科技创新本质上是人的创造性活动。要充分尊重和信任科研人员，赋予创新团队和领军人才更大的人财物支配权和技术路线决策权。进一步提高基础研究项目间接经费占比，开展项目经费使用“包干制”改革试点，不设科目比例限制，由科研团队自主决定使用。完善科技成果评价机制。要在推动科技体制改革举措落地见效上下功夫，决不能让改革政策停留在口头上、纸面上。大力简除烦苛，使科研人员潜心向学、创新突破。加强科研伦理和学风建设，惩戒学术不端，力戒浮躁之风。我国有世界上最大规模的科技人才队伍，营造良好的科研生态，就一定能够迎来各类英才竞现、创新成果泉涌的生动局面。

进一步把大众创业万众创新引向深入。鼓励更多社会主体创新创业，拓展经济社会发展空间，加强全方位服务，发挥双创示范基地带动作用。强化普惠性支持，落实好小规模纳税人增值税起征点从月销售额3万元提高到10万元等税收优惠政策。改革完善金融支持机制，设立科创板并试点注册制，鼓励发行双创金融债券，扩大知识产权质押融资，支持发展创业投资。改革完善人才培养、使用、评价机制，优化归国留学人员和外籍人才服务。把面向市场需求和弘扬人文精神结合起来，善聚善用各类人才，中国创新一定能更好发展，为人类文明进步作出应有贡献。

(四)促进形成强大国内市场，持续释放内需潜力。充分发挥消费的基础作用、投资的关键作用，稳定国内有效需求，为经济平稳运行提供有力支撑。

推动消费稳定增长。多措并举促进城乡居民增收，增强消费能力。落实好新修订的个人所得税法，使符合减税政策的约8000万纳税人应享尽享。要顺应消费需求的新变化，多渠道增加优质产品和服务供给，加快破除民间资本进入的堵点。我国60岁以上人口已达2.5亿。要大力发展养老特别是社区养老服务业，对在社区提供日间照料、康复护理、助餐助行等服务的机构给予税费减免、资金支持、水电气热价格优惠等扶持，新建居住区应配套建设社区养老服务设施，加强农村养老服务设施建设，改革完善医养结合政策，扩大长期护理保险制度试点，让老年人拥有幸福的晚年，后来人就有可期的未来。婴幼儿照护事关千家万户。要针对实施全面两孩政策后的新情况，加快发展多种形式的婴幼儿照护服务，支持社会力量兴办托育服务机构，加强儿童安全保障。促进家政服务业提质扩容。发展全域旅游，壮大旅游产业。稳定汽车消费，继续执行新能源汽车购置优惠政策，推动充电、加氢等设施建设。发展消费新业态新模式，促进线上线下消费融合发展，培育消费新增长点。健全农村流通网络，支持电商和快递发展。加强消费者权益保护，让群众放心消费、便利消费。

合理扩大有效投资。紧扣国家发展战略，加快实施一批重点项目。完成铁路投资8000亿元、公路水运投资1.8万亿元，再开工一批重大水利工程，加快川藏铁路规划建设，加大城际交通、物流、市政、灾害防治、民用和通用航空等基础设施投资力度，加强新一代信息基础设施建设。今年中央预算内投资安排5776亿元，比去年增加400亿元。创新项目融资方式，适当降低基础设施等项目资本金比例，用好开发性金融工具，吸引更多民间资本参与重点领域项目建设。落实民间投资支持政策，有序推进政府和社会资本合作。改革完善招投标制度。政府要带头讲诚信守契约，决不能“新官不理旧账”，对拖欠企业的款项年底前要清偿一半以上，决不允许增加新的拖欠。

(五)对标全面建成小康社会任务，扎实推进脱贫攻坚和乡村振兴。坚持农业农村优先发展，加强脱贫攻坚与乡村振兴统筹衔接，确保如期实现脱贫攻坚目标、农民生活达到全面小康水平。

打好精准脱贫攻坚战。重点解决实现“两不愁三保障”面临的突出问题，加大“三区三州”等深度贫困地区脱贫攻坚力度，加强基础设施建设，落实对特殊贫困人口的保障措施。脱贫致富离不开产业支撑，要大力扶持贫困地区发展特色优势产业。开展贫困地区控辍保学专项行动、明显降低辍学率，继续增加重点高校专项招收农村和贫困地区学生人数，用好教育这个阻断贫困代际传递的治本之策。基本完成“十三五”易地扶贫搬迁规划建设任务，加强后续扶持。对摘帽县和脱贫人口的扶持政策要保持一段时间，巩固脱贫成果。完善考核监督，用好中央脱贫攻坚专项巡视成果。越是到脱贫攻坚的关键阶段，越要抓实抓细各项工作，确保脱贫有实效、可持续、经得起历史检验。

抓好农业特别是粮食生产。近14亿中国人的饭碗，必须牢牢端在自己手上。要稳定粮食产量，优化品种结构。加强农田水利建设，新增高标准农田8000万亩以上。稳定生猪等畜禽生产，做好非洲猪瘟等疫病防控。加快农业科技改革创新，大力发展现代种业，加强先进实用技术推广，实施地理标志农产品保护工程，推进农业全程机械化。培育家庭农场、农民合作社等新型经营主体，加强面向小农户的社会化服务，发展多种形式规模经营。扶持主产区发展农产品精深加工。支持返乡入乡创业创新，推动一二三产业融合发展，壮大县域经济。务工收入是农民增收的大头。要根治拖欠农民工工资问题，抓紧制定专门行政法规，确保付出辛劳和汗水的农民工按时拿到应有的报酬。

扎实推进乡村建设。科学编制和实施建设规划，大力改善生产生活条件。加快实施农村饮水安全巩固提升工程，今明两年要解决好饮水困难人口的饮水安全问题，提高6000万农村人口供水保障水平。完成新一轮农村电网升级改造。新建改建农村公路20万公里。继续推进农村危房改造。因地制宜开展农村人居环境整治，推进“厕所革命”、垃圾污水治理，建设美丽乡村。

全面深化农村改革。推广农村土地征收、集体经营性建设用地入市、宅基地制度改革试点成果。深化集体产权、集体林权、国有林区林场、农垦、供销社等改革。改革完善农业支持保护体系，健全粮食价格市场化形成机制，扩大政策性农业保险改革试点，创新和加强农村金融服务。持续深化农村改革，广袤乡村必将焕发新的生机活力。

(六)促进区域协调发展，提高新型城镇化质量。围绕解决发展不平衡不充分问题，改革完善相关机制和政策，促进基本公共服务均等化，推动区域优势互补、城乡融合发展。

优化区域发展格局。制定西部开发开放新的政策措施，西部地区企业所得税优惠等政策到期后继续执行。落实和完善促进东北全面振兴、中部地区崛起、东部率先发展的改革创新举措。京津冀协同发展重在疏解北京非首都功能，高标准建设雄安新区。落实粤港澳大湾区发展规划纲要，促进规则衔接，推动生产要素流动和人员往来便利化。将长三角区域一体化发展上升为国家战略，编制实施发展规划纲要。长江经济带发展要坚持上中下游协同，加强生态保护修复和综合交通运输体系建设，打造高质量发展经济带。支持资源型地区经济转型。加快补齐革命老区、民族地区、边疆地区、贫困地区发展短板。大力发展蓝色经济，保护海洋环境，建设海洋强国。

深入推进新型城镇化。坚持以中心城市引领城市群发展。抓好农业转移人口落户，推动城镇基本公共服务覆盖常住人口。更好解决群众住房问题，落实城市主体责任，改革完善住房市场体系和保障体系，促进房地产市场平稳健康发展。继续推进保障性住房建设和城镇棚户区改造，保障困难群体基本居住需求。继续推进地下综合管廊建设。城镇老旧小区量大面广，要大力进行改造提升，更新水电路气等配套设施，支持加装电梯和无障碍环境建设，健全便民市场、便利店、步行街、停车场等生活服务设施。新型城镇化要处处体现以人为核心，提高柔性化治理、精细化服务水平，让城市更加宜居，更具包容和人文关怀。

(七)加强污染防治和生态建设，大力推动绿色发展。绿色发展是构建现代化经济体系的必然要求，是解决污染问题的根本之策。要改革完善相关制度，协同推动高质量发展与生态环境保护。

持续推进污染防治。巩固扩大蓝天保卫战成果，今年二氧化硫、氮氧化物排放量要下降3%，重点地区细颗粒物(PM2.5)浓度继续下降。持续开展京津冀及周边、长三角、汾渭平原大气污染治理攻坚，加强工业、燃煤、机动车三大污染源治理。做好北方地区清洁取暖工作，确保群众温暖过冬。强化水、土壤污染防治，今年化学需氧量、氨氮排放量要下降2%。加快治理黑臭水体，防治农业面源污染，推进重点流域和近岸海域综合整治。加强固体废弃物和城市垃圾分类处置，促进减量化、资源化、无害化。加强污染防治重大科技攻关。企业作为污染防治主体，必须依法履行环保责任。改革创新环境治理方式，对企业既依法依规监管，又重视合理诉求、加强帮扶指导，对需要达标整改的给予合理过渡期，避免处置措施简单粗暴、一关了之。企业有内在动力和外部压力，污染防治一定能取得更大成效。

壮大绿色环保产业。坚持源头治理，加快火电、钢铁行业超低排放改造，实施重污染行业达标排放改造。调整优化能源结构。推进煤炭清洁化利用。健全天然气产供储销体系。大力发展可再生能源，加快解决风、光、水电消纳问题。加大城市污水管网和处理设施建设力度。促进资源节约集约和循环利用，推广绿色建筑、绿色快递包装。改革完善环境经济政策，健全排污权交易制度，加快发展绿色金融，培育一批专业化环保骨干企业，提升绿色发展能力。

加强生态系统保护修复。推进山水林田湖草生态保护修复工程试点，持续抓好国土绿化，加强荒漠化、石漠化、水土流失治理。加大生物多样性保护力度。继续开展退耕还林还草还湿。深化国家公园体制改革。健全生态补偿机制。绿色发展人人有责，贵在行动、成在坚持。我们要共同努力，让人民群众享有美丽宜居环境。

(八)深化重点领域改革，加快完善市场机制。聚焦突出矛盾和关键环节，推动相关改革深化，健全与高质量发展相适应的体制机制，把市场活力和社会创造力充分释放出来。

加快国资国企改革。加强和完善国有资产监管，推进国有资本投资、运营公司改革试点，促进国有资产保值增值。积极稳妥推进混合所有制改革。完善公司治理结构，健全市场化经营机制，建立职业经理人等制度。依法处置“僵尸企业”。深化电力、油气、铁路等领域改革，自然垄断行业要根据不同行业特点实行网运分开，将竞争性业务全面推向市场。国有企业要通过改革创新、强身健体，不断增强发展活力和核心竞争力。

下大气力优化民营经济发展环境。坚持“两个毫不动摇”，鼓励、支持、引导非公有制经济发展。按照竞争中性原则，在要素获取、准入许可、经营运行、政府采购和招投标等方面，对各类所有制企业平等对待。构建亲清新型政商关系，健全政企沟通机制，激发企业家精神，促进民营经济发展升级。保护产权必须坚定不移，对侵权行为要依法惩处，对错案冤案要有错必纠。要努力打造良好营商环境，让企业家安心搞经营、放心办企业。

深化财税金融体制改革。加大预算公开改革力度，全面实施预算绩效管理。深化中央与地方财政事权和支出责任划分改革，推进中央与地方收入划分改革。完善转移支付制度。健全地方税体系，稳步推进房地产税立法。规范地方政府举债融资机制。以服务实体经济为导向，改革优化金融体系结构，发展民营银行和社区银行。改革完善资本市场基础制度，促进多层次资本市场健康稳定发展，提高直接融资特别是股权融资比重。增强保险业风险保障功能。加强金融风险监测预警和化解处置。我国财政金融体系总体稳健，可运用的政策工具多，我们有能力守住不发生系统性风险的底线。

(九)推动全方位对外开放，培育国际经济合作和竞争新优势。进一步拓展开放领域、优化开放布局，继续推动商品和要素流动型开放，更加注重规则等制度型开放，以高水平开放带动改革全面深化。

促进外贸稳中提质。推动出口市场多元化。扩大出口信用保险覆盖面。改革完善跨境电商等新业态扶持政策。推动服务贸易创新发展，引导加工贸易转型升级、向中西部转移，发挥好综合保税区作用。优化进口结构，积极扩大进口。办好第二届中国国际进口博览会。加快提升通关便利化水平。

加大吸引外资力度。进一步放宽市场准入，缩减外资准入负面清单，允许更多领域实行外资独资经营。落实金融等行业改革开放举措，完善债券市场开放政策。加快与国际通行经贸规则对接，提高政策透明度和执行一致性，营造内外资企业一视同仁、公平竞争的公正市场环境。加强外商合法权益保护。赋予自贸试验区更大改革创新自主权，增设上海自贸试验区新片区，推进海南自贸试验区建设、探索建设中国特色自由贸易港。支持国家级经开区、高新区、新区开展自贸试验区相关改革试点，增强辐射带动作用，打造改革开放新高地。中国投资环境一定会越来越好，各国企业在华发展机遇一定会越来越多。

推动共建“一带一路”。坚持共商共建共享，遵循市场原则和国际通行规则，发挥企业主体作用，推动基础设施互联互通，加强国际产能合作，拓展第三方市场合作。办好第二届“一带一路”国际合作高峰论坛。推动对外投资合作健康有序发展。

促进贸易和投资自由化便利化。中国坚定维护经济全球化和自由贸易，积极参与世贸组织改革。加快构建高标准自贸区网络，推进区域全面经济伙伴关系协定、中日韩自贸区、中欧投资协定谈判，继续推动中美经贸磋商。中国秉持互利合作、共赢发展，一贯主张通过平等协商解决贸易争端。我们对作出的承诺认真履行，对自身合法权益坚决维护。

(十)加快发展社会事业，更好保障和改善民生。今年财政收支平衡压力加大，但基本民生投入确保只增不减。支持社会力量增加非基本公共服务供给，满足群众多层次、多样化需求。

发展更加公平更有质量的教育。深化教育教学改革。推进城乡义务教育一体化发展，加快改善乡村学校办学条件，加强乡村教师队伍建设，抓紧解决城镇学校“大班额”问题，保障进城务工人员随迁子女教育，发展“互联网+教育”，促进优质资源共享。多渠道扩大学前教育供给，无论是公办还是民办幼儿园，只要符合安全标准、收费合理、家长放心，政府都要支持。推进高中阶段教育普及，办好民族教育、特殊教育、继续教育，依法支持民办教育发展。持续抓好义务教育教师工资待遇落实。推进一流大学和一流学科建设，支持中西部建设有特色、高水平大学。今年财力虽然很紧张，国家财政性教育经费占国内生产总值比例继续保持在4%以上，中央财政教育支出安排超过1万亿元。我们要切实把宝贵的资金用好，努力办好人民满意的教育，托起明天的希望。

保障基本医疗卫生服务。继续提高城乡居民基本医保和大病保险保障水平，居民医保人均财政补助标准增加30元，一半用于大病保险。降低并统一大病保险起付线，报销比例由50%提高到60%，进一步减轻大病患者、困难群众医疗负担。加强重大疾病防治。我国受癌症困扰的家庭以千万计，要实施癌症防治行动，推进预防筛查、早诊早治和科研攻关，着力缓解民生的痛点。做好常见慢性病防治，把高血压、糖尿病等门诊用药纳入医保报销。加快儿童药物研发。加强罕见病用药保障。深化医保支付方式改革，优化医保支出结构。抓紧落实和完善跨省异地就医直接结算政策，尽快使异地就医患者在所有定点医院能持卡看病、即时结算，切实便利流动人口和随迁老人。完善药品集中采购和使用机制。深化公立医院综合改革。促进社会办医。发展“互联网+医疗健康”，加快建立远程医疗服务体系，加强基层医疗卫生机构能力建设和医护人员培养，提升分级诊疗和家庭医生签约服务质量。坚持预防为主，将新增基本公共卫生服务财政补助经费全部用于村和社区，务必让基层群众受益。抓好传染病、地方病、青少年近视防治。完善生育配套政策，加强妇幼保健服务。支持中医药事业传承创新发展。加强健康教育和健康管理。药品疫苗攸关生命安全，必须强化全程监管，对违法者要严惩不贷，对失职渎职者要严肃查办，坚决守住人民群众生命健康的防线。

完善社会保障制度和政策。推进多层次养老保障体系建设。继续提高退休人员基本养老金。落实退役军人待遇保障，完善退役士兵基本养老、基本医疗保险接续政策。适当提高城乡低保、专项救助等标准，加强困境儿童保障。加大城镇困难职工脱困力度。提升残疾预防和康复服务水平。我们要尽力为群众救急解困、雪中送炭，基本民生的底线要坚决兜牢。

丰富人民群众精神文化生活。培育和践行社会主义核心价值观，广泛开展群众性精神文明创建活动，大力弘扬奋斗精神、科学精神、劳模精神、工匠精神，汇聚起向上向善的强大力量。加快构建中国特色哲学社会科学。加强互联网内容建设。繁荣文艺创作，发展新闻出版、广播影视和档案等事业。加强文物保护利用和非物质文化遗产传承。推动文化事业和文化产业改革发展，提升基层公共文化服务能力。倡导全民阅读，推进学习型社会建设。深化中外人文交流。广泛开展全民健身活动。扎实做好2020年奥运会、残奥会备战工作，精心筹办北京冬奥会、冬残奥会，办好第七届世界军人运动会。人民群众身心健康，社会就充满活力，国家就繁荣兴旺。

加强和创新社会治理。推动社会治理重心向基层下移，推广促进社会和谐的“枫桥经验”，构建城乡社区治理新格局。引导支持社会组织、人道救助、志愿服务和慈善事业健康发展。健全社会信用体系。保障妇女、儿童、老人、残疾人合法权益。改进信访工作，依法及时解决群众合理诉求。加强社会心理服务。健全国家应急体系，提高防灾减灾救灾能力。加强安全生产，防范遏制重特大事故。做好地震、气象、水文、地质、测绘等工作。健全公共法律服务体系，深化普法宣传教育。加强国家安全能力建设。完善立体化社会治安防控体系，深入推进扫黑除恶专项斗争，依法惩治盗抢骗黄赌毒等违法犯罪活动，打击非法集资、传销等经济犯罪，整治侵犯公民个人信息等突出问题，坚决守护好人民群众的平安生活。

各位代表!

新的形势和任务，对政府工作提出了新的更高要求。各级政府要树牢“四个意识”，坚定“四个自信”，坚决做到“两个维护”，自觉在思想上政治上行动上同以习近平同志为核心的党中央保持高度一致，落实全面从严治党要求，勇于自我革命，深入推进简政放权，加快转职能、提效能，增强政府公信力和执行力，更好满足人民对美好生活的新期待。

坚持依法全面履职。深入贯彻全面依法治国基本方略，严格遵守宪法法律，把政府活动全面纳入法治轨道。各级政府要依法接受同级人大及其常委会的监督，自觉接受人民政协的民主监督，主动接受社会和舆论监督，让权力在阳光下运行。政府干的，都应是人民盼的。要坚持科学、民主、依法决策，认真听取人大代表、政协委员意见，听取民主党派、工商联、无党派人士和各人民团体意见，听取社会公众和企业意见，使各项政策符合基本国情和客观实际，更接地气、更合民意。全面推进政务公开。支持工会、共青团、妇联等群团组织更好发挥作用。全面落实行政执法责任制和问责制，对一切违法违规的行为都要坚决查处，对一切执法不公正不文明的现象都要坚决整治，对所有行政不作为的人员都要坚决追责。

深入推进党风廉政建设。扎实开展“不忘初心、牢记使命”主题教育。认真贯彻落实中央八项规定及其实施细则精神，持之以恒纠治“四风”。加强廉洁政府建设，一体推进不敢腐、不能腐、不想腐。强化审计监督。政府工作人员要自觉接受法律监督、监察监督和人民监督。衡量政绩最终是看结果。各级政府要坚决反对和整治一切形式主义、官僚主义，让干部从文山会海、迎评迎检、材料报表中解脱出来，把精力用在解决实际问题上。压减和规范督查检查考核事项，实施“互联网+督查”。减少开会和发文数量，今年国务院及其部门要带头大幅精简会议、坚决把文件压减三分之一以上。

切实强化责任担当。中国改革发展的巨大成就，是广大干部群众筚路蓝缕、千辛万苦干出来的。实现“两个一百年”奋斗目标，成就中国人民的幸福与追求，还得长期不懈地干。为政以公，行胜于言。各级政府及其工作人员要求真务实、力戒浮华，以推动改革发展的成果说话，以干事创业的实绩交卷。健全激励约束机制和尽职免责机制，营造干部愿干事、敢干事、能干成事的环境。更好发挥中央和地方两个积极性，尊重基层和群众首创精神，为地方大胆探索提供激励、留足空间。广大干部要树立强烈的事业心和进取心，事不避难、义不逃责，埋头苦干、结合实际创造性地干，努力干出无愧于人民的新业绩，干出中国发展的新辉煌。

各位代表!

我们要坚持和完善民族区域自治制度，全面贯彻党的民族政策，深化民族团结进步教育，铸牢中华民族共同体意识，促进各民族和睦相处、和衷共济、和谐发展。加大对民族地区和人口较少民族发展的支持，深入实施兴边富民行动，同心协力建设56个民族共同团结奋斗、共同繁荣发展的美好家园。

我们要全面贯彻党的宗教工作基本方针，坚持我国宗教的中国化方向，依法管理宗教事务，发挥宗教界人士和信教群众在促进经济社会发展中的积极作用。

我们要认真落实侨务政策，保障海外侨胞和归侨侨眷合法权益，改善和加强服务，发挥好他们的独特优势和重要作用，画好海内外中华儿女的最大同心圆，汇聚起共创辉煌的澎湃力量。

各位代表!

过去一年，国防和军队建设扎实推进，强军事业展现许多新气象新作为。新的一年，要继续以党在新时代的强军目标为引领，牢固确立习近平强军思想在国防和军队建设中的指导地位，深入推进政治建军、改革强军、科技兴军、依法治军。坚持党对军队绝对领导的根本原则和制度，全面深入贯彻军委主席负责制。贯彻新时代军事战略方针，提高实战化军事训练水平，坚决维护国家主权、安全、发展利益。继续深化国防和军队改革，建立健全中国特色社会主义军事政策制度体系。加强和完善国防教育、国防动员体系建设，增强全民国防意识。深入实施军民融合发展战略，加快国防科技创新步伐。各级政府要大力关心支持国防和军队建设，深入开展“双拥”活动，让军政军民团结之树根深叶茂、永葆常青。

各位代表!

我们要继续全面准确贯彻“一国两制”、“港人治港”、“澳人治澳”、高度自治的方针，严格依照宪法和基本法办事。全力支持香港、澳门特别行政区政府和行政长官依法施政。支持港澳抓住共建“一带一路”和粤港澳大湾区建设的重大机遇，更好发挥自身优势，全面深化与内地互利合作。我们坚信，香港、澳门一定能与祖国内地同发展共进步、一定能保持长期繁荣稳定。

我们要坚持对台工作大政方针。全面贯彻落实习近平总书记在《告台湾同胞书》发表40周年纪念会上的重要讲话精神，坚持一个中国原则和“九二共识”，推动两岸关系和平发展、推进祖国和平统一进程。坚决反对和遏制“台独”分裂图谋和行径，坚决维护国家主权和领土完整。深化两岸融合发展，持续扩大两岸经济文化交流合作。两岸同胞同根相系、同命相连，应携手共创共享全体中国人的美好未来。

各位代表!

当今世界面临百年未有之大变局。我们将坚定不移走和平发展道路、奉行互利共赢的开放战略，坚定维护多边主义和以联合国为核心的国际体系。积极参与全球治理体系的改革完善，坚定维护开放型世界经济，推动构建人类命运共同体。加强与主要大国沟通对话与协调合作，深化同周边国家关系，拓展与发展中国家互利合作。积极为妥善应对全球性挑战和解决地区热点问题提供更多中国建设性方案。中国愿与各国携手合作、同舟共济，为促进世界持久和平与共同发展作出新的贡献。

各位代表!

奋斗创造历史，实干成就未来。我们要更加紧密地团结在以习近平同志为核心的党中央周围，高举中国特色社会主义伟大旗帜，以习近平新时代中国特色社会主义思想为指导，迎难而上，开拓进取，以经济社会发展的优异成绩迎接中华人民共和国成立70周年，为决胜全面建成小康社会、夺取新时代中国特色社会主义伟大胜利，为把我国建设成为富强民主文明和谐美丽的社会主义现代化强国、实现中华民族伟大复兴的中国梦不懈奋斗!
